# Endogenous growth and the influence of information and communication technology on Poland's economic trajectory

**DOI:** 10.12688/openreseurope.19945.1

**Published:** 2025-05-12

**Authors:** J.S. Keshminder, Maciej Woźniak, Rafal Kusa, Abdul Rahim Ridzuan, Naila Erum

**Affiliations:** 1 Department of Economics and Financial Studies, Universiti Teknologi MARA, Shah Alam, Selangor, Malaysia, 40450, Malaysia; 2 Faculty of Management, AGH University, Gramatyka 10, Kraków, Poland; 3 5Accounting Research institute, University teknologi MARA, Shah Alam, Selangor, 40450, Malaysia; 4 Komplex Al-Khawarizmi, Universiti Teknologi MARA (UiTM), Institute of Big Data Analytics and Artificial Intelligence, Shah Alam, Selangor, 40450, Malaysia

**Keywords:** Information and communication technology, Poland, Innovation, Endogenous economic growth, Economic growth.

## Abstract

**Background:**

Poland has experienced a significant digital revolution, driven by technological advancements and supportive government initiatives. The increased use and integration of information and communication technology (ICT) have played a crucial role in this transformation. Understanding the economic impacts of these changes is essential, particularly through indicators such as gross fixed capital formation, labour force dynamics, human capital and education, technology/innovation, foreign direct investment (FDI), and ICT infrastructure.

**Method:**

This research employs data spanning 33 years, from 1990 to 2022, to explore the relationship between ICT and economic growth in Poland. The Autoregressive Distributed Lag (ARDL) bounds testing methodology is used to derive empirical results and assess both short- and long-term dynamics.

**Results:**

The findings indicate that ICT, labour, and FDI have a positive and significant impact on Poland’s economic growth, with labour exerting the most substantial influence. Conversely, capital investment demonstrates a negative effect on economic growth, likely due to inefficiencies in allocation and diminishing returns in certain sectors.

**Conclusion:**

Based on the results, several key policy recommendations are proposed to further enhance Poland's economic growth. This research also contributes to the macroeconomic theory of endogenous growth by providing new insights into the role of ICT and associated factors in an emerging digital economy.

## Plain Language Summary

Poland has seen a dramatic digital revolution spurred by technological breakthroughs and supportive government initiatives. The growing use of information and communication technology (ICT) has been vital for greater economic progress. This research provides a comprehensive understanding of the economic impact of ICT in Poland by looking at essential indicators like gross fixed capital formation, labour force dynamics, human capital and education, technology/innovation, foreign direct investment, and ICT infrastructure. Thirty-three years of data from 1990 to 2022 using sound economic techniques was used for analysis. The empirical results indicated that ICT, labour, and FDI positively and significantly impact Poland's economic growth, with labour having the most decisive influence. Based on the findings, several key policy recommendations are presented to enhance Poland's economic development.

## Introduction

Over the past few decades, Poland, a fast-rising country in Central Europe, has experienced substantial economic transformations. The growing use and incorporation of information and communication technology (ICT) have been vital to this shift. ICT stands for information and communication technologies, which are essential to current economic activity since they enable connectivity, information processing, and communication ( Gerow *et al.*, 2015). Endogenous growth theory identifies ICT as a critical component impacting Poland's economic trajectory, highlighting the relevance of internal variables like human capital, innovation, and knowledge spillovers in driving economic progress ( Etro, 2023; Nash *et al.*, 2023).

Even with Poland's significant digitalisation progress, in-depth research is still required to pinpoint precisely how ICT has impacted the country's economic expansion. Although many studies have examined how ICT affects economic growth internationally and in other locations, more research must be done in Poland ( Turap *et al.*, 2019). Previous studies frequently focus on more general economic aspects rather than thoroughly examining the unique contributions that digital technology has made to Poland's economy. By thoroughly examining the connection between ICT and economic growth in Poland, this study seeks to close this knowledge gap and provide insightful information for stakeholders and policymakers.

According to the studies, Doru *et al.* (2022), Kovács *et al.* (2023), and Masoura & Malefaki (2023), Poland's digital transformation has advanced significantly, as seen by the country's rising internet penetration, increasing mobile subscriber rates, and extensive use of digital services. Poland has demonstrated notable advancements in connectivity, digital skills, and the incorporation of digital technologies in enterprises, per the 2023 edition of the Digital Economy and Society Index (DESI). Still, there are obstacles to overcome, especially in closing the digital gap and improving the workforce's digital competencies.

By offering a detailed examination of how Poland's economic growth has been impacted by digitalisation, this study adds to the body of current material. This research provides a comprehensive understanding of the economic impact of ICT in Poland by looking at essential indicators like gross fixed capital formation, labour force dynamics, human capital and education (tertiary school enrolment rates), technology/innovation (patents per capita), foreign direct investment (FDI), and ICT infrastructure (fixed telephone subscriptions). This study attempts explicitly to (1) Evaluate the Effect of ICT on Economic Growth, (2) Calculate the ICT sector's contributions to Poland's total economic development and GDP growth, (3) Determine the Main Drivers and Obstacles: Emphasise the primary drivers of digital adoption as well as the obstacles that must be removed to realise the economic benefits of ICT fully. (4) Provide policymakers practical advice on supporting Poland's transition to a more robust and inclusive digital economy.

Poland must comprehend the relationship between ICT and economic growth as it becomes a more inventive and digital economy. The study's insights can help guide strategic decisions about investing in human capital, improving digital infrastructure, and creating an atmosphere encouraging innovation and technical developments. This research aims to increase Poland's competitiveness and economic resilience in the global digital economy.

## Poland's digital trajectory

Over the past ten years, Poland has seen a dramatic digital revolution spurred by technological breakthroughs and supportive government initiatives. According to Eurostat (2023), the percentage of Poles who use the Internet increased from 65% in 2010 to over 90% in 2022. This indicates a steady increase in internet usage in the country. This trend is credited with the growth of broadband infrastructure and the rising affordability of internet services. One of the main factors improving digital connectivity is the widespread use of cell phones. As of 2023, over 80% of Poles were utilising mobile internet services, indicating a significant increase in mobile internet subscriptions ( Kemp, 2023). More people can now access digital services and information, which has led to increased digital inclusion.

When it comes to digitising public services, the Polish government has made significant strides. An example of this initiative is the launch of the ePUAP platform, which gives individuals online access to several government services. By 2023, almost all government services were offered online, greatly enhancing citizen pleasure and administrative effectiveness. Poland's digital economy has expanded quickly, primarily because of the e-commerce industry. In the last five years, online retail sales have doubled; by 2022, they will account for 14% of all retail sales. The spread of digital payment methods and customer confidence in online buying are the main drivers of this rise. Poland has made significant investments in the development of 5G networks as well as its ICT infrastructure. The introduction of 5G technology is anticipated to improve connection and foster the expansion of the Internet of Things (IoT) applications, spurring additional innovation in the digital space.

Poland's digitalisation has changed the country drastically in several economic areas, fostering greater creativity, productivity, and economic expansion. Digitalisation has increased Poland's GDP growth considerably. According to a report by Kozuń-Cieślak & Markowska-Bzducha (2021) In Poland, the digital economy made up 15% of the country's GDP in 2021; estimates indicate that percentage would rise to 20% by 2025. The increase in e-commerce, digital services, and the ICT sector are the main drivers of this growth. There are now more job prospects due to the digital transformation, especially in the tech and digital services industries. However, it has also made a change in the skill sets necessary, with a focus on technical and digital literacy. The Polish government has recently launched programs to upskill workers, such as collaborations with the commercial sector and digital training efforts.

A growing number of tech firms in sectors including fintech, edtech, and health tech are sprouting in Poland, where the startup ecosystem has grown. Among other supportive policies, venture capital and R&D tax incentives have made innovation and entrepreneurship more accessible. Public service delivery has changed due to digitalisation, which is becoming more accessible and efficient. Especially during the COVID-19 epidemic, the quality and accessibility of healthcare, education, and government services have increased with digital platforms. Polish firms have rapidly embraced digital technologies to improve their operations. Digital tools and platforms are widely employed in marketing, supply chain management, and customer interaction to increase efficiency and competitiveness.

Noteworthy developments and profound effects on the whole economy have marked Poland's digitisation ( Małkowska *et al.*, 2021). Maintaining this pace and ensuring that the advantages of digitalisation are widely distributed throughout society will require ongoing investment in ICT infrastructure, digital skill development, and supportive policy ( Kowalski & Weresa, 2019).

## Endogenous growth theory and its components

A significant development in economic theory is the endogenous growth model, which emphasises the role of internal variables rather than external influences in causing economic growth. The endogenous growth model asserts that endogenous variables like human capital, innovation, and knowledge spillovers are primarily responsible for economic growth, in contrast to the exogenous growth model, which credits external factors like technological advancements for long-term economic progress.

Capital investment in tangible assets such as machinery, equipment, and infrastructure lays the foundation for endogenous growth theory. It is known as gross fixed capital formation (GFCF) and is essential to production capacity ( Elfaki & Ahmed, 2024). According to endogenous growth theory, these investments boost productive capacity and support long-term economic growth ( Etro, 2023). For example, a recent study by de Clercq *et al.* (2023) supported the findings of De Long and Summers' 2019 study, which shows a positive relationship between GFCF and economic growth and confirms that higher investment rates spur higher production—additionally Adamczyk and Ilik (2022) revealed that both public and private infrastructure investment potentially accelerates economic growth in both rich and developing nations. Baran (2013) states that the capital contribution to the economic growth was very significant during the transition (1995 – 2010) of Central Europe countries, including Poland, in contrast to labour. Inessa *et al*. (2019) state that there was a correlation between the gross expenditure on tangible assets and investment growth rate of both tangible and current assets of enterprises and the economics growth in Poland The time series was, however, quite short (2005–2017) and they did not take the time lags into consideration. These finding was supported by Baran (2023) or Woźniak *et al*. (2021). The former study was conducted by Granger causality and the best time lag of delay was three years. Skare *et al.* (2019) reveal that the most important impact on GDP in Poland had total factor productivity and human capital stock both in short and long run.

The labour force within the endogenous growth theory emphasises the vital need for human resources for production and economic development, especially quality labour for economic progress. Past and present studies have consistently supported the positive link between human capital and economic growth ( Kowalski & Weresa, 2019). Kowalski & Weresa (2019) implied that, if accompanied by complementary efforts to promote labour productivity, demographic changes can result in significant economic advantages and increases in the working-age population.

In addition to the labour force, human capital through education and training expenditures increases labour productivity, leading to economic expansion. The endogenous economic growth theory proves that education expenditures produce a more knowledgeable and efficient workforce. Current studies often show the importance of postsecondary education for economic expansion. Hanushek & Wößmann (2010) advocated the link between excellent tertiary enrolment rates and faster economic growth—education has the property to stimulate a productive and innovative workforce. Economies depending on knowledge-intensive industries recorded superior growth with higher investment in higher education ( Kowalski & Weresa, 2019). Hanushek & Wößmann (2010) further justified Lucas's, 1988 study finding that human capital buildup is essential for long-term economic prosperity.

Foreign direct investment (FDI) is frequently researched in the context of the endogenous growth theory. Empirical evidence shows that FDI can foster economic growth by assisting economies by transferring capital, management expertise, technology, and innovation ( Turap *et al.*, 2019). Additionally, Kowalski & Weresa (2019) discovered that nations with higher human capital levels have more substantial growth effects from FDI, proving a complementary relationship between FDI and homegrown capacity.

Technology and innovation are also often engaged through the endogenous growth model to see their impact on economic growth—new processes, goods, and efficiency produced by innovation stimulate economic growth. Technological progress is an internal process businesses acquire via research and development, leading to innovations and knowledge spillovers for sustained economic progress. Undeniably, many current and past studies have found a strong correlation between technology/innovation and economic growth ( Baran, 2023; de Clercq *et al.*, 2023; Turap *et al.*, 2019; Zamparelli, 2024).

Infrastructure related to information and communication technology (ICT), such as fixed phone subscriptions, is essential for economic expansion. Modern economies depend on ICT because it improves communication, lowers transaction costs, and makes information more accessible ( Kowalski & Weresa, 2019). Recent research emphasises the role that ICT plays in economic expansion. Neamtu *et al.* (2021) Prove that by facilitating better coordination and communication, telecommunications infrastructure—including fixed telephone lines—significantly accelerates economic growth. Additionally, Kovács *et al.* (2023) it was discovered that better economic growth rates correlate with more use of ICT, especially in emerging nations where this infrastructure is still growing.

Endogenous growth offers a strong foundation for comprehending the internal forces propelling economic expansion. According to recent empirical research, the labour force, human capital and education, technology and innovation, foreign direct investment, and information and communications technology (ICT) all play essential roles in fostering sustainable economic growth. Enhancing these elements through policy can have significant long-term economic advantages.

## Methods

This study investigates the macroeconomic determinants for endogenous growth model for Polland with main focus on ICT. The period of the studies is based on 33 years of observation starting from 1990 to 2022. The sources of all variables are from the World Development Indicators (WDI) (see Table 1). The details of the variables and its proxy is shown in the table below. The data used in this study is available in the Sample Dataset for ESG Analysis [1], accessible via Zenodo at https://zenodo.org/records/13836845 ( Jit Singh & Hanif, 2024). The data was analysed using EViews software ( IHS Markit, 2021).

**Table 1.** Sources of data.

Variable	Proxy
GDP represent economic growth	GDP per capita (constant 2015 US$)
CAP represent capital	Gross fixed capital formation (% of GDP)
LAB represent labor	Total Labor force
HC represent human capital	School enrollment, tertiary (% gross)
TECH represent technology	(Patent applications, nonresidents + Patent applications, residents) / Total Population
FDI represent foreign direct investment	Foreign direct investment, net inflows (% of GDP)
ICT represent internet and telecommunication technology	Fixed telephone subscriptions (per 100 people)

Note: The sources of the data is derived from WDI (2024)

The basic regression model is adapted from endogenous growth model as specified in Equation 1,


GDP=f(CAP,LAB,HC)(Equation1)

The model as shown in Equation 1 is extended with more additional macroeconomic variables]. To ensure the robustness of the analysis, all variables are expressed in their natural logarithms, which serves to normalize the data and mitigate potential heteroscedasticity. The new equation as follows:


LNGDP=LNCAP,LNLAB,LNHC,LNTECH,LNFDI,LNICT(Equation2)

To meet the research objectives, this study employs the Autoregressive Distributed Lag (ARDL) bounds testing methodology, as introduced by Pesaran *et al.* (2001). The ARDL approach is particularly suitable for small sample sizes, providing reliable results even with limited data ( Narayan, 2004). Moreover, it does not require prior knowledge of whether the variables are integrated at level or first difference, making it flexible for analyzing variables with mixed integration orders. Additionally, ARDL can detect multiple cointegrating relationships between variables ( Nkoro & Uko, 2016). Given the sample size of 33 observations and variables integrated at levels and first differences, but not at second differences, the ARDL approach is especially well-suited for this study.

The ARDL regression model is specified in Equation 3, where △ denotes the first difference operator and ε _t_ represents a white-noise disturbance term. The equation is devided into two parts: the first part captures the short-run dynamics, while the second part reflects the long-run relationships.


$$\begin{array}{l} \Delta LNGDP_{t}=\beta_{0}+\theta_{0}LNGDP_{t-1}+\theta_{1}LNCAP_{t-1}+\theta_{2}LNLAB_{t-1}+\theta_{3}LNHC_{t-1}\\ \;+\theta_{4}LNTECH_{t-1}+\theta_{5}LNFDI_{t-1}+\theta_{6}LNICT_{t-1}+\sum_{i=1}^{a}\beta_{i}\Delta LNGDP_{t-i}\\ \;+\sum_{i=0}^{b}\gamma_{i}\Delta LNCAP_{t-i}+\sum_{i=0}^{c}\lambda_{i}\Delta LNLAB_{t-i}+\sum_{i=0}^{d}\vartheta_{i}\Delta LNHC_{t-i}\\ \;+\sum_{i=0}^{d}\psi_{i}\Delta LNTECH_{t-i}+\sum_{i=0}^{f}\delta_{i}\Delta LNFDI_{t-i}+\sum_{i=0}^{g}\delta_{i}\Delta LNICT_{t-i}+\upsilon_{t} \end{array}\;(\text{Equation}\;3)$$

To ensure the robustness of the ARDL methodology, thorough preliminary testing is crucial. This begins with stationarity testing, a key requirement for accurate time series analysis. Unit root tests are vital in this context, as they help detect non-stationarity and prevent misleading regression results ( Shrestha & Bhatta, 2006). Non-stationary variables can distort data properties, leading to unreliable and spurious regressions. In this study, the Augmented Dickey-Fuller (ADF) and Phillips-Perron (PP) tests will be employed to assess the presence of a unit root.

Following this, several diagnostic tests will be conducted to evaluate the model's goodness of fit. The Lagrange Multiplier Serial Correlation (LMSC) test will check for serial correlation, where error terms across observations might be correlated, potentially biasing OLS estimates and leading to inefficient standard errors. The Breusch-Pagan-Godfrey (BPG) test will assess heteroscedasticity, a condition where error variance changes across observations. Although heteroscedasticity does not bias coefficient estimates, it can affect standard error efficiency and undermine hypothesis testing. The Ramsey Regression Equation Specification Error Test (RESET) will be applied to identify potential specification errors, such as omitted variables, irrelevant variables, or incorrect functional forms. Residual normality will be assessed through skewness and kurtosis to ensure they follow a normal distribution. Lastly, the Cumulative Sum (CUSUM) and Cumulative Sum of Squares (CUSUMSQ) tests will evaluate the stability of the model's parameters over time, using recursive residual charts to test for consistency in parameter estimates throughout the sample.

## Results

Before explaining the short-run and long-run elasticities, a series of preliminary tests were conducted. The first analysis involved Unit Root tests, as presented in Table 2. The stationarity of the variables was assessed using two common tests: the Augmented Dickey-Fuller (ADF) test and the Phillips-Perron (PP) test. These tests were performed to determine the stationarity of each variable at their level, I(0), and at their first difference, I(1). The results confirmed that the variables exhibit mixed stationarity at both I(0) and I(1) levels, thereby validating the use of the ARDL (Autoregressive Distributed Lag) model to investigate the short-run and long-run relationships among the variables.

**Table 2.** Unit Root Test.

Level I(0)	ADF Unit Root		PP Unit Root	
	Intercept	Intercept and Trend	Intercept	Intercept and Trend
LNGDP	0.718 (0)	-3.426 (0) *	0.718 (0)	-3.783 (3) **
LNCAP	-3.313 (1) **	-3.168 (3)	-2.075 (1)	-1.966 (3)
LNLAB	-0.653 (0)	-1.898 (0)	-0.653 (0)	-1.902 (1)
LNHC	-2.978 (1) **	-1.624 (7)	-3.534 (2) **	-0.508 (2)
LNTECH	-0.457 (0)	-2.179 (1)	-0.640 (2)	-1.834 (2)
LNFDI	-4.165 (0) ***	-4.008 (0) **	-4.998 (11) ***	-4.157 (8) **
LNICT	-2.180 (1)	-1.978 (1)	-2.054 (3)	-1.430 (0)
First difference I(1)	ADF Unit Root		PP Unit Root	
	Intercept	Intercept and Trend	Intercept	Intercept and Trend
LNGDP	-4.261 (1) ***	-3.567 (8) *	-6.727 (3) ***	-6.791 (5) ***
LNCAP	-3.508 (4) *	-4.565 (4) ***	-2.808 (6) *	-2.861 (8)
LNLAB	-5.208 (0) ***	-5.175 (0) ***	-5.209 (1) ***	-5.171 (2) ***
LNHC	-2.609 (0)	-3.870 (2) **	-2.485 (1)	-4.035 (5) **
LNTECH	-4.317 (0) ***	-4.331 (0) ***	-4.306 (2) ***	-4.342 (1) ***
LNFDI	-6.874 (0) ***	-6.864 (0) ***	-7.725 (14) ***	-8.770 (19) ***
LNICT	-2.688 (0) *	-3.450 (0) *	-2.728 (1) *	-3.389 (3) *

Note: 1. ***, ** and * are 1%, 5% and 10% of significant levels, respectively. 2. The optimal lag length is selected automatically using the Schwarz Info Criteria (SIC) for ADF test and the bandwidth had been selected by using the Newey–West method for PP unit root test.

The long-run cointegration test was conducted using the F-statistic. The results, presented in Table 3, indicate that with a maximum lag of (2,3) and a lag order of (2,2,3,3,3,3), the F-statistic recorded a value of 8.276, which is significant at the 1% level. This probability value exceeds the critical value of the upper bound at the 1% significance level, confirming the presence of a long-run cointegrating relationship in the model.

**Table 3.** Long run cointegration based on F stat.

Model	Max Lag	Lag order	F statistics
LNGDP = f(LNCAP, LNLAB, LNHC, LNTECH, LNFDI, LNICT)	(2,3)	(2, 2, 3, 3, 3, 3, 3)	8.276 ***
Critical Values for F stat		Lower I(0)	Upper (1)
10%		2.12	3.23
5%		2.45	3.61
1%		3.15	4.43

Note: 1. # The critical values are based on Pesaran *et al.* (2001), case III: unrestricted intercept and no trend. 2. k is a number of variables and it is equivalent to 5. 3. *, **, and *** represent 10%, 5% and 1% level of significance, respectively.

**Figure 1.**
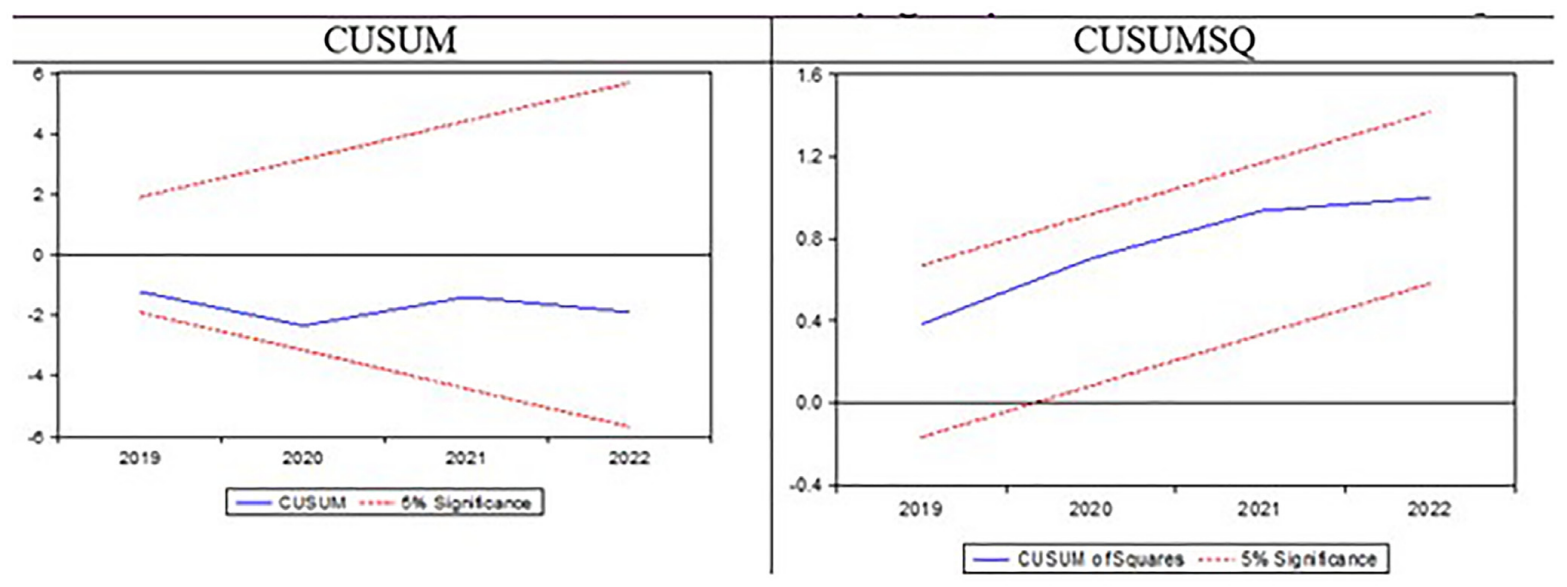
CUSUM and CUSUM SQ test.

Before examining the long-run and short-run elasticities, a series of diagnostic tests were performed to ensure the reliability of the model. Table 4 and Figure 1 summarizes five types of diagnostic tests: the serial correlation test, functional form test, normality test, heteroscedasticity test, and stability test based on the CUSUM and CUSUM SQ tests. The results indicate that all diagnostic tests for the proposed model show no issues, as their probability values are greater than the 10% significance level. Additionally, the model is considered stable, as the blue dotted line in both the CUSUM and CUSUM SQ tests lies within the two dotted red lines as shown in Figure 1.

**Table 4.** Diagnostic Tests.

Model	(A)	(B)	(C)	(D)
	Serial Correlation [p-value]	Functional Form [p-value]	Normality [p-value]	Heteroscedasticity [p-value]
LNGDP = f(LNCAP, LNLAB, LNHC, LNTECH, LNFDI, LNICT)	3.251 [0.235]	0.995 [0.391]	0.276 [0.871]	3.699 [0.105]

Note. 1. ** represents 5% significant levels. 2. The diagnostic test performed as follows A. Lagrange multiplier test for residual serial correlation; B. Ramsey’s RESET test using the square of the fitted values; C. Based on a test of skewness kurtosis of residuals; D. Based on the regression of squared fitted values. 2.

The results of the short-run elasticities are presented in Table 5. First, considering the current lag, it is shown that LNLAB has a positive and significant relationship with LNGDP for Poland. Statistically, a 1% increase in LNLAB leads to a 2.118% increase in LNGDP. Additionally, LNTECH, LNFDI, and LNICT also exhibit positive and significant relationships with LNGDP. Specifically, a 1% increase in LNTECH, LNFDI, and LNICT results in an increase in economic growth by 0.015%, 0.06%, and 0.21%, respectively. The error correction term (ECT) indicates a significant and negative sign, with a coefficient of -0.25. This implies that 25% of any short-run disequilibrium is corrected each period, leading the model to converge to its long-run equilibrium over time. This result is a crucial indicator for policy recommendations.

**Table 5.** Short-run elasticities and error correction model.

Variables	Coefficient	t-stat	Prob
ΔLNGDP _(-1)_	-0.123	-0.752	0.493
ΔLNCAP	0.057	0.855	0.440
ΔLNCAP _(-1)_	0.114	1.498	0.208
ΔLNLAB	2.118	4.171	0.014
ΔLNLAB _(-1)_	-0.883	-1.557	0.194
ΔLNLAB _(-2)_	1.004	1.630	0.178
ΔLNHC	-0.105	-0.954	0.393
ΔLNHC _(-1)_	-0.204	-2.133	0.099
ΔLNHC _(-2)_	-0.226	-3.275	0.030
ΔLNTECH	0.015	2.379	0.076
ΔLNTECH _(-1)_	0.019	1.329	0.254
ΔLNTECH _(-2)_	-0.034	-3.616	0.022
ΔLNFDI	0.060	5.688	0.004
ΔLNFDI _(-1)_	-0.036	-4.900	0.008
ΔLNFDI _(-2)_	-0.043	-5.858	0.004
ΔLNICT	0.210	3.039	0.038
ΔLNICT _(-1)_	-0.467	-3.760	0.019
ΔLNICT _(-2)_	0.272	3.511	0.024
ECT _(-1)_	-0.251	-3.407	0.027

The main outcomes for the long run are presented in Table 6. Among the selected variables, only LNCAP, LNLAB, and LNFDI have a significant relationship with the dependent variable. Notably, higher capital leads to lower economic growth in Poland. Statistically, a 1% increase in LNCAP results in a 0.771% decrease in economic growth. This counterintuitive result could be due to several factors: inefficiencies in capital allocation, diminishing returns on capital investments, or an over-reliance on capital-intensive industries that do not foster sustainable growth. It might also reflect the saturation of capital investments in certain sectors, leading to reduced marginal productivity of additional capital.

**Table 6.** Long-run elasticities.

Variables	Coefficient	t-stat	Prob
LNCAP	-0.771	-2.250	0.087
LNLAB	12.077	4.940	0.007
LNHC	-0.056	-0.256	0.810
LNTECH	0.024	0.694	0.525
LNFDI	0.587	4.567	0.010
LNICT	-0.038	-0.247	0.816
C	-190.357	-4.732	0.009

Conversely, LNLAB and LNFDI have positive and significant effects on economic growth. A 1% increase in LNLAB leads to a 12.077% increase in economic growth, highlighting the importance of labor in driving economic performance. Similarly, a 1% increase in LNFDI results in a 0.587% increase in economic growth, underscoring the beneficial impact of foreign direct investment on the economy. These findings suggest that while capital investment may currently have adverse effects, focusing on labor and attracting foreign investment can be more effective strategies for fostering economic growth in Poland.

Table 6 summarizes the main outcomes. Among the selected variables, only LNCAP, LNLAB, and LNFDI exhibit significant relationships with the dependent variable. Higher capital (LNCAP) leads to lower economic growth in Poland. A 1% increase in LNCAP results in a 0.771% decrease in economic growth.

## Discussion

We found that there was a negative impact of LNCAP on the economic growth in Poland, which is in opposition to the previous studies. This counterintuitive result can be attributed to several factors. Inefficiencies in capital allocation may arise when investments are directed toward less productive sectors, leading to suboptimal economic outcomes ( Cerra & Saxena, 2008). Moreover, diminishing returns on capital can occur, where increasing the capital stock does not yield proportional economic benefits ( Gancia & Zilibotti, 2009). Over-reliance on capital-intensive sectors, such as heavy industry, which have traditionally received substantial investment in Poland, may have reached a saturation point, reducing the marginal productivity of additional capital ( Kremer, 1993). The negative relationship between capital and economic growth may be due to structural imbalances within the Polish economy. Over-investment in particular sectors can reduce the marginal productivity of capital ( Pritchett, 2000). Additionally, if capital investments are funded by high levels of debt, the financial burden could stifle long-term economic growth ( Panizza & Presbitero, 2014). One need to take into consideration the turmoil of Covid-19 and war in Ukraine. Although new in investments in capital in the years 2020–2022 were conducted, there was a decrease in GDP because of implemented restrictions of economic activity. That might have explain the negative impact of LNCAP on GDP. In contrast, both LNLAB and LNFDI exhibit positive and significant effects on economic growth. A 1% increase in LNLAB leads to a 12% increase in economic growth, underscoring the crucial role of labor in driving economic performance. This is consistent with studies emphasizing the significance of human capital and labor productivity for economic growth ( Acemoglu & Autor, 2011). Similarly, a 1% increase in LNFDI results in a 0.6 % rise in economic growth. Foreign direct investment not only contributes capital but also transfers technology, managerial expertise, and productivity improvements ( Borensztein *et al.*, 1998).

In the long term, the sustained positive effects of labor and foreign direct investment can be attributed to workforce improvements, enhanced productivity, and the influx of new technologies and managerial practices through foreign investments. Since Poland’s accession to the European Union in 2004, the country has attracted significant FDI, which has driven technological upgrades and managerial expertise ( Javorcik, 2004).

## Conclusion

This study highlights the significant role that Information and Communication Technologies (ICT) have played in shaping Poland’s economic growth trajectory. The findings highlight the positive contributions of ICT, labor, and foreign direct investment (FDI) to Poland’s GDP, particularly in the tech and digital services sectors. While ICT has proven to be a catalyst for economic development in short run, challenges such as the digital divide and inadequate workforce skills persist. This research provides a comprehensive understanding of the economic impact of ICT in Poland by looking at essential indicators like gross fixed capital formation, labour force dynamics, human capital and education, technology/innovation, FDI, and ICT infrastructure. For this purpose we used data of 33 years starting from 1990 to 2022 and the Autoregressive Distributed Lag (ARDL) bounds testing methodology is applied to compile empirical results. The empirical results reveal that ICT, labor, and FDI have positive and significant impacts on Poland’s economic growth, with labor having the strongest influence. However, capital investment exhibits a negative relationship with economic growth, likely due to inefficiencies in allocation and diminishing returns in certain sectors.

Based on the findings, several key policy recommendations are essential to enhance Poland's economic growth through the effective use of Information and Communication Technologies (ICT), labor, and Foreign Direct Investment (FDI). First, labor force development and upskilling should be prioritized, as labor (LNLAB) has a strong positive impact on GDP both in short and long run. Increasing investments in education, vocational training, and promoting STEM fields will equip the workforce with the necessary skills for the digital economy. Additionally, attracting FDI is crucial, given its role in transferring technology and managerial expertise. Policymakers should offer fiscal incentives and ease regulatory barriers to encourage foreign investments in key sectors like ICT and innovation.

The study also highlights inefficiencies in capital allocation (LNCAP), which negatively impacts economic growth in long run. Policymakers should reform capital allocation to direct investments toward high-growth sectors such as digital services, rather than capital-intensive industries. Reducing debt-driven capital investments will also ensure long-term financial sustainability.

Strengthening ICT infrastructure is another priority, as a 1% increase in ICT adoption leads to a 0.21% rise in GDP. Expanding broadband access, particularly in rural areas, and fostering public-private partnerships for digital innovation will help drive further economic gains.

Furthermore, promoting technological advancement through increased R&D spending and better intellectual property protection will accelerate innovation in Poland. Offering for patents and innovation will also incentivize technological progress. Efforts should be made to ensure inclusive digital transformation by bridging the digital divide through nationwide digital literacy programs, with special attention to marginalized groups and regions. Support for small and medium-sized enterprises (SMEs) in adopting digital technologies is equally important for enhancing their competitiveness.

Finally, stabilizing short-run economic adjustments is necessary to manage volatility. Countercyclical fiscal policies, such as increasing public spending during downturns, and a flexible monetary policy will support the economy’s short-term needs while sustaining long-term growth. In conclusion, these policies will enable Poland to capitalize on its digital transformation, ensuring sustainable and inclusive economic growth.

The study has, however, some constraints, too. First of all, the significance level is quite high in case of gross fixed capital formation impact on economic growth. Therefore, one should consider the result with caution. Moreover, we do not know whether the negative impact of LNCAP is caused by private or public entities. Therefore, more research is needed. One need to study the determinants of GDP without the impact of turmoil of Covid-19 pandemic or war in Ukraine.

## Ethics and consent

Ethical approval and consent were not required.

## Data Availability

Zenodo: Circular Economy, Digitalization and Environment. Doi: https://doi.org/10.5281/zenodo.13836845 ( Jit Singh & Hanif, 2024) This project contains the following underlying data: WDIEXCEL 2024 poland malaysia.xlsx Data is available under the terms of the Creative Commons Attribution 4.0 International

## Software availability statement

EViews software was used in this study. A free trial version of EViews (Student Version) can be accessed through the following website: https://www.eviews.com/download/student11/.
