# Supplementary material for: Endogenous growth and the influence of information and communication technology on Poland's economic trajectory
Source: Open Res Eur. 2025 May 12;5:131. [Version 1] doi: 10.12688/openreseurope.19945.1 (PMC12397740; doi:10.12688/openreseurope.19945.1)
Supplement: Peer review history

# Reviewer report: Reviewer response for version 1

**DOI:** 10.21956/openreseurope.21574.r56361

**Published:** 2025-08-29

**Authors:** Sutrisno Sutrisno

**Affiliations:** 1 Universitas PGRI Semarang, Jawa Tengah, Indonesia

The manuscript is well-written. The introduction is clearly explained, and the research's novelty is evident. The researcher has used appropriate methods that support the answer to the research question. The reviewer suggests the use of the skewness and kurtosis tests, as these are robust tests based on expert opinion (see  reference). The results section is clearly presented in each subsection. However, this is not the case in the discussion section. The discussion is considered very shallow. A deeper discussion of the factors driving and inhibiting economic growth in Poland should be provided. The limitations of this study should also be addressed. This will provide a comprehensive research landscape for readers, researchers pursuing this topic, or stakeholders in policymaking.

If applicable, is the statistical analysis and its interpretation appropriate?

Yes

Is the study design appropriate and is the work technically sound?

Yes

Is the work clearly and accurately presented and does it engage with the current literature?

Yes

Are the conclusions drawn adequately supported by the results?

Yes

Are sufficient details of methods and analysis provided to allow replication by others?

Yes

Are all the source data and materials underlying the results available?

Yes

Reviewer Expertise:

mathematics; statistics; education

I confirm that I have read this submission and believe that I have an appropriate level of expertise to confirm that it is of an acceptable scientific standard.

## References

[1] : Mardia’s Skewness and Kurtosis for Assessing Normality Assumption in Multivariate Regression. *Enthusiastic : International Journal of Applied Statistics and Data Science* .2021; 10.20885/enthusiastic.vol1.iss1.art1 1-6 10.20885/enthusiastic.vol1.iss1.art1

# Reviewer report: Reviewer response for version 1

**DOI:** 10.21956/openreseurope.21574.r56356

**Published:** 2025-08-21

**Authors:** Karafolas Simeon

**Affiliations:** 1 University of Western Macedonia, Koila Kozanis, Greece

The paper examines an interesting area on the specific case of Poland. It presents an originality.

 Generally, it is well structured. The literature provided is sufficient and quite updated. 

 Method analysis is well structured and well interpreted and conclusions supported the results.

 Nevertheless, some issues must be added at the paper.

 •    Poland, like other Eastern European countries, emerged from an economic disaster at the beginning of the '90s. Authors could provide a comparative table or graph of the evolution of the Polish GDP and the consolidated GDP of European Union countries to illustrate differences in development. Are there different periods of development?

 •    Authors could provide data on the parameters considered in this paper in the appendix.

 •    The authors mention the ePUAP platform: they must explain it.

 •    Authors mention the consequences of the COVID-19 epidemic (ex., Page 5 left column); they must be more specific on this period, for example, mentioning studies referring to economic consequences during this period in Poland. The same for the global financial crisis of 2008. 

 •    Authors mention the counterintuitive result between capital and growth of GDP; they mention some reasons; they may also examine an eventual delay between capital investments and results that should appear. Examine, or at least mention studies, if other factors such as COVID-19 pandemic or Ukraine war or, earlier, global financial crisis, prevented the GDP growth and, consequently, they cancelled to a certain extent the positive results that the capital investments would have had. For this reason, it is important to have data on the evolution of GDP growth during the whole period examined, as well as any study referring to specific periods.

 •    On results, (page 8 right column), authors mention a sectoral issue, (“saturation of capital investments in certain sectors”); could they be more specific on which sectors they refer?

If applicable, is the statistical analysis and its interpretation appropriate?

Yes

Is the study design appropriate and is the work technically sound?

Yes

Is the work clearly and accurately presented and does it engage with the current literature?

Yes

Are the conclusions drawn adequately supported by the results?

Yes

Are sufficient details of methods and analysis provided to allow replication by others?

Yes

Are all the source data and materials underlying the results available?

Partly

Reviewer Expertise:

NA

I confirm that I have read this submission and believe that I have an appropriate level of expertise to confirm that it is of an acceptable scientific standard, however I have significant reservations, as outlined above.

# Reviewer report: Reviewer response for version 1

**DOI:** 10.21956/openreseurope.21574.r55997

**Published:** 2025-08-20

**Authors:** Pasrun Adam

**Affiliations:** 1 Universitas Halu Oleo, Kendari, Indonesia

After reading the manuscript entitled "Endogenous Growth and the Influence of Information and Communication Technology on Poland's Economic Trajectory," some comments are as follows:

 1. The introduction fully presents the background, research gaps, and research objectives. However, the formulation of the objectives should fully state all independent variables that influence the dependent variable.

 2. In the methods section, equation 2 is still incorrectly written. The correct form of equation 2 is shown in Tables 3 and Table 4.

 3. The independent variables: labor and human capital, as well as technology and ICT, have the potential to be highly correlated. It is recommended to report the results of the multicollinearity test.

 4. The discussion section should also include policy recommendations for the government of Polland related to the research findings.

If applicable, is the statistical analysis and its interpretation appropriate?

Yes

Is the study design appropriate and is the work technically sound?

Yes

Is the work clearly and accurately presented and does it engage with the current literature?

Yes

Are the conclusions drawn adequately supported by the results?

Yes

Are sufficient details of methods and analysis provided to allow replication by others?

Partly

Are all the source data and materials underlying the results available?

Yes

Reviewer Expertise:

Economics

I confirm that I have read this submission and believe that I have an appropriate level of expertise to confirm that it is of an acceptable scientific standard.
